# Identification and Biological Activity of Synthetic Macrophage Inducible C-Type Lectin Ligands

**DOI:** 10.3389/fimmu.2017.01940

**Published:** 2018-01-17

**Authors:** Chriselle D. Braganza, Thomas Teunissen, Mattie S. M. Timmer, Bridget L. Stocker

**Affiliations:** ^1^School of Chemical and Physical Sciences, Victoria University of Wellington, Wellington, New Zealand; ^2^Centre for Biodiscovery, Victoria University of Wellington, Wellington, New Zealand

**Keywords:** C-type lectin, Mincle, pathogen-associated molecular pattern, damage-associated molecular pattern, adjuvant, glycolipid

## Abstract

The macrophage inducible C-type lectin (Mincle) is a pattern recognition receptor able to recognize both damage-associated and pathogen-associated molecular patterns, and in this respect, there has been much interest in determining the scope of ligands that bind Mincle and how structural modifications to these ligands influence ensuing immune responses. In this review, we will present Mincle ligands of known chemical structure, with a focus on ligands that have been synthetically prepared, such as trehalose glycolipids, glycerol-based ligands, and 6-acylated glucose and mannose derivatives. The ability of the different classes of ligands to influence the innate, and consequently, the adaptive, immune response will be described, and where appropriate, structure–activity relationships within each class of Mincle ligands will be presented.

## Introduction

Macrophage inducible C-type (calcium-dependent) lectin (Mincle, Clec4e, ClecSf9) is a pattern recognition receptor that is involved in the innate immune response to pathogen-associated molecular patterns (PAMPs) and damage-associated molecular patterns (DAMPs). First identified in 1999 by Matsumoto et al. as a downstream target of the nuclear factor (NF) that binds the interleukin (IL)-1 responsive element in the IL-6 gene (1), there has been much subsequent interest in elucidating the structure of exogenous and endogenous Mincle ligands. Mincle has been shown to sense dead cells through a protein component of small nuclear ribonucleoprotein (SAP130) (2) and to recognize yeast (e.g., *Candida albicans*) (3, 4) and fungi (e.g., *Malassezia*) (5); however, the first defined non-proteinaceous Mincle ligand was identified in 2009 by Ishikawa et al. and found to be trehalose dimycolate (TDM) (6), the most abundant glycolipid in the cell wall of *Mycobacterium tuberculosis*. In the same year, Werninghaus et al. determined that C-type lectins, rather than toll like receptors (TLRs), recognize TDM (7), with the same group later demonstrating that Mincle is essential for the recognition and adjuvanticity of TDM and its related C22 linear analog, trehalose dibehenate (TDB) (8).

**Figure 1:**
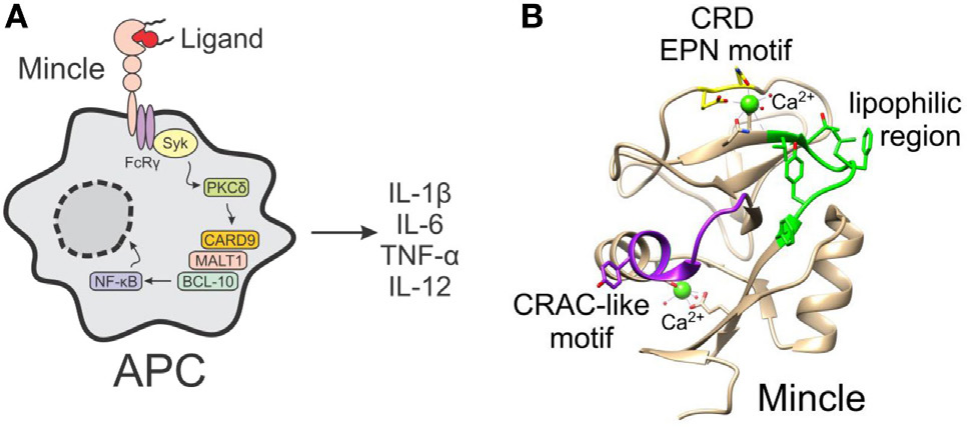
**(A)** The activation of macrophage inducible C-type lectin (Mincle) on antigen-presenting cells by ligands leads to the induction of the FcRγ-Syk-Card9 pathway and NF-κB-mediated gene expression. **(B)** Crystal structure of human Mincle (18), with the carbohydrate recognition domain EPN motif indicated in yellow, the lipophilic region in green, and the cholesterol recognition/interaction amino acid consensus-like motif in purple.

In humans and rodents, Mincle is expressed on a variety of cell types of the myeloid lineage (e.g., monocytes, macrophages, neutrophils, and dendritic cells) (1, 2, 9, 10) and some subsets of B cells (11), with the binding and subsequent activation of Mincle by ligands leading to the activation of the FcRγ-Syk-Card9-dependent pathway and NF-κB-mediated gene expression (Figure 1A) (2, 6–8). This activation results in the expression of many different inflammatory genes, ranging from cytokines (e.g., TNF-α, IL-6, IL-1β, and IL-12), to various chemokines and enzymes generating small molecule mediators like iNOS. Ultimately, these cellular mediators influence the adaptive immune response and T-helper cell differentiation. Moreover, the type of Mincle ligand can affect the ensuing immune response, and this has deemed Mincle agonists to be promising vaccine adjuvants (12–15) in the same way that TLR ligands show much potential in this respect (16, 17).

Insight into the binding site of Mincle ligands has gone some way into understanding how the different classes of Mincle ligand may bind to the receptor. Mincle shares high homology with other carbohydrate binding proteins (lectins), with the crystal structure of human Mincle (hMincle) and bovine Mincle (bMincle) being elucidated in 2013 by Furukawa et al. (18) and Feinberg et al. (19), respectively. The carbohydrate recognition domain (CRD) of Mincle (Figure 1B) is comprised of the common EPN motif (residues 169–171), which is often observed in C-type (calcium-dependent) lectin receptors (CLRs) and which has been shown to be indispensable for TDM recognition. Mincle uses this Ca^2+^ ion to bind the equatorial 3- and 4-OH groups of one glucose residue in trehalose (18, 19). An additional secondary binding site lacking Ca^2+^ accommodates the second glucose moiety of the trehalose disaccharide and this extra recognition provides increased binding affinity for trehalose by 36-fold compared to glucose (19). Moreover, Arg183 of hMincle is crucially involved in ligand recognition (18), and is in a suitable position to interact with hydroxyl groups on TDMs.

The regions surrounding the Ca^2+^ site in Mincle, however, are distinct from those in other CLRs. A series of hydrophobic pockets are found in Mincle, but not other CLRs, with the hydrophobic region of hMincle being composed of Val195, Thr196, Phe198, Leu199, Tyr201, and Phe202, which gives rise to an open-side groove to one side of the primary sugar binding site (18). Similarly, in bMincle, a shallow hydrophobic grove was also observed adjacent to the sugar binding site—albeit, to the other side of the EPN motif (19). As there is no crystal structure with bound TDM or related glycolipid, the exact mode of lipid binding is unknown; however, the aforementioned hydrophobic groove is the proposed recognition site for one of the lipids (18, 19). In addition to the major hydrophobic grove, there is also a second minor hydrophobic groove in Mincle, which is thought to be able to accommodate the second lipid in TDM (18, 19). When comparing hMincle and mouse Mincle (mMincle), there are subtle differences between the two species, however, on the whole, the two lectin domains contain highly identical folds and there is 85% sequence homology between the species (1). Notwithstanding, for certain classes of ligand (such as glycerol esters, *vide infra*), some Mincle species-specific activity is observed.

In addition to the carbohydrate-binding domain, Mincle also contains a second binding site, which has been shown to recognize DAMPs (2, 20). Less is known about Mincle binding of DAMPs; however, Yamasaki et al. suggested that SAP130 binds to a site other than the EPN motif (2), while Kiyotake et al. demonstrated that cholesterol crystals bind to hMincle *via* a cholesterol recognition/interaction amino acid consensus (CRAC)-like motif (Figure 1B) (20). Thus, it appears that Mincle has several binding domains that can accommodate a variety of PAMPs and DAMPs.

Several groups have used knowledge of the Ca^2+^ CRD of Mincle to better design Mincle ligands, or to understand how known Mincle ligands might be accommodated by the receptor (as described below). That said, it is not clear if Mincle binding alone correlates to a functional immune response. Thus, while knowledge of the Mincle binding site can serve to guide the design of potential Mincle agonists, functional studies whereby a variety of Mincle ligands are screened for their ability to activate the innate immune response are required in order to better identify promising Mincle ligands. Accordingly, this review focuses not only on the different classes of ligands that can bind and activate Mincle but also on the known structure-activity requirements for each class of ligands.

## Trehalose Diesters (TDEs)

### Trehalose Dimycolates (TDMs)

**Figure 2:**
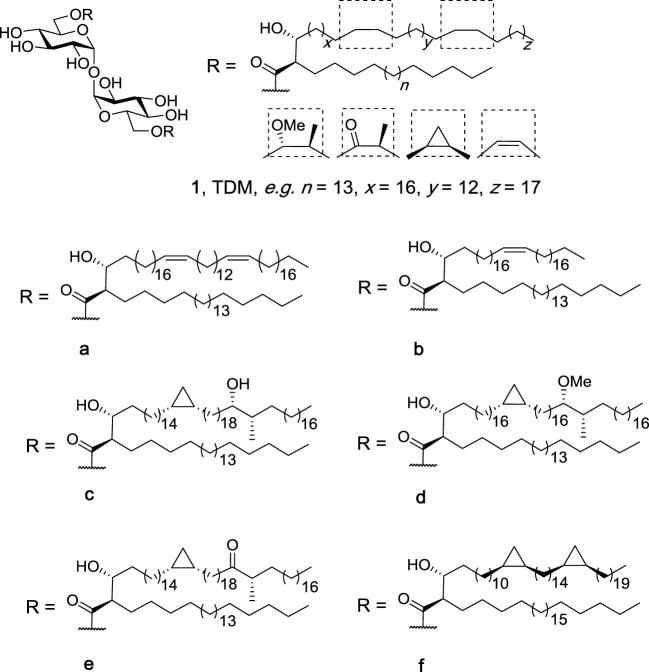
Isolated trehalose dimycolate (TDM) (**1**) from natural sources, such as *Mycobacterium tuberculosis*, consists of a mixture of up to 500 different structures with a variety of side-chain functional groups and different lipid lengths (*n, x, y*, and *z*). Structures **1a–f** are representative homogenous TDMs synthesized by Baird et al. (27, 28).

Trehalose dimycolates (**1**, Figure 2), historically known as cord factor (21, 22), are the most abundant glycolipids in the cells wall of *Mycobacterium* species (23, 24). TDMs consist of an α,α′-trehalose core with the 6- and 6′-position esterified to mycolic acid which contain at least two chiral centers: an alkyl chain at the α-position and a hydroxyl group at the β-position, both with the *R*-configuration (25). The mycolic acid can be of varying length and contains functional groups consisting of either oxygenated mycolates (methoxy-, keto-, and epoxy-mycolates) or non-oxygenated mycolates (*cis*-cyclopropane, alkenes, and dienes) (25). In 1950, Bloch showed that cord factor was capable of activating the immune system (21), and since then, there has been much interest in understanding how the structural features of TDM relate to the immunomodulatory properties of the individual molecules (26, 27). To date, more than 500 closely related structures of TDM have been identified (25).

While the immunostimulatory activity of TDMs has been known for some time, Mincle was only recently identified as a receptor for TDMs. In 2009, Yamasaki et al. (6) used nuclear factor of activated T cells-green fluorescent protein (NFAT-GFP) reporter cells expressing Mincle to determine that Mincle is responsible for the direct recognition of TDM, and the simplified TDM analog TDB. Using Mincle deficient mice, Yamasaki et al. demonstrated that bone marrow-derived macrophages (BMMs) were no longer capable of producing tumor necrosis factor (TNF) and macrophage inflammatory protein 2 (MIP-2) when exposed to TDM and TDB, suggesting that this process is Mincle dependent. Yamasaki et al. also showed that this activity required the EPN motif, as recognition of TDM was eliminated in mutant Mincle^EPN→QPD^ NFAT-GFP reporter cells (6).

Simultaneously, Werninghaus et al. used Syk^−/−^, Card9^−/−^, and FcRγ^−/−^ BMMs to determine that both TDM and TDB activate macrophages through an FcRγ-Syk-Card9-dependent pathway (7), with further studies by the Lang group showing that this process is both Mincle and FcRγ dependent (8).

The complexity of TDMs has meant that only a few groups have been able to synthesize and tease apart the immunomodulatory properties of the individual glycolipids. In 1978, Parant et al. (29) isolated mycolic acids ranging from C80 to C90 in length from mycobacteria as well as low-molecular-weight mycolic acids (C28 to C36) from *Corynebacterium diphtheria* and coupled these to trehalose. Immunization of mice with the different TDMs followed by infection with *Klebsiella pneumoniae* or *Lysteria monocytogenes* demonstrated that the low-molecular-weight mycolic acids were found to be equally potent at protecting the mice against infection as their longer-chain counterparts. However, as mixtures of compounds containing a variety of lipid lengths and functional groups were ultimately tested in each case, detailed structure-activity relationships could not be derived.

Insight into the potential of the individual TDMs to lead to differences in the immune response was first possible due to the elegant synthetic endeavors of Baird et al. (28, 30–38). Initially, Al Dulayymi et al. synthesized a library of mycolic acids (28), whereby a key step in the total syntheses was the use of a Fráter–Seebach alkylation to install the α-branch and the β-hydroxyl group in the correct *anti*-configuration. The mycolic acids were then selectively coupled to the 6- and 6′-positions of α,α′-trehalose to give 16 TDMs (**1a**–**f**), including those containing diene (**1a**), alkene (**1b**), hydroxyl (**1c**), methoxy (**1d**), and keto (**1e**) functional groups in the meromycolate branch (28). In a first series of studies, the individual TDMs, as well as a mixture of isolated TDM and synthetic TDB, were tested for their ability to activate bone marrow-derived dendritic cells (BMDCs), as measured by the production of TNF-α (27). Here, it was shown that the diene TDM (**1a**), which lacks the two cyclopropane groups, had a lower inflammatory potential compared to isolated TDM, but induced similar levels of TNF-α compared to TDB. Moreover, the methoxy-functionalized TDM (**1d**) led to higher levels of TNF-α compared to the hydroxy-functionalized TDM (**1c**) suggesting that increased polarity and the potential for hydrogen bonding in the middle of the meromycolate branch does not increase binding and activation of Mincle. As the *cis*-methoxy TDM (**1d**) was found to induce similar levels of TNF-α compared to that of isolated TDM, this material was chosen as the lead compound.

Subsequent studies with the *cis-*methoxy TDM (**1d**) determined that this homogenous material caused BMDCs to induce similar levels of IL-6, IL-1β, and IL-12P40 to that produced by BMDCs in response to isolated TDM (27). Moreover, *cis-*methoxy TDM (**1d**) and isolated TDM both upregulated the surface expression of costimulatory molecules CD86, CD80, and MHC-II, with the immune response to TDM, TDB, and synthetic TDM (**1d**) being Mincle, FcRy, and Malt1 dependent. Synthetic TDM (**1d**) was also found to activate the NLRP3 inflammasome in a manner similar to isolated TDM, and *in vivo* studies, where mice were injected in the footpad with TDM (**1d**), isolated TDM or TDB as an emulsion [30% incomplete freunds adjuvant (IFA), glycolipid (10 μg/mouse), ovalbumin (50 μg/mouse)], showed that all three trehalose glycolipids caused similar footpad swelling, infiltration of granulocytes (Ly6C^+^ and Ly6G^+^), and cytokine and chemokine production (IL-1β, IL-12p35, TNF-α, and CXCL1).

### Trehalose Dicorynomycolates (TDCMs)

**Figure 3:**
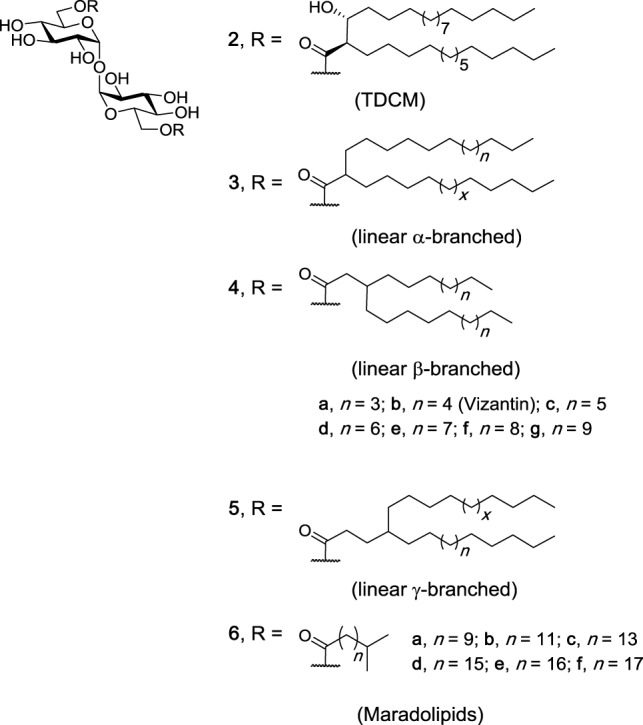
Other branched trehalose diesters (TDEs). trehalose dicorynomycolate (TDCM) (**2**), first isolated from *Corynebacterium* species, occurs naturally as a mixture of chain lengths. Various TDCMs and dehydroxy derivatives have been synthesized, as have the maradolipids, which have been first isolated from the dauer larva stage of *Caenorhabditis elegans*. Of these branched TDEs, TDCM (**1**) and maradolipids (**6a–f**) have been confirmed to be macrophage inducible C-type lectin agonists (42, 43, 47, 48).

Like TDM, TDCMs (e.g., **2**, Figure 3) consist of a trehalose core esterified at the 6- and 6′-positions, however instead of the more complex mycolic acids, TDCMs are esterified with corynomycolic acids, which consist of a shorter α-branch and a hydroxyl at the β-position, with both substituents in the *R* configuration (39). TDCMs were first isolated from *Corynebacteria diptheriae* in 1963 and were found to have carbon lengths ranging from C24 to C32 (40). Since this time, studies have demonstrated that TDCMs activate macrophages and cause tumor regression (41–43) and that TDCMs are less toxic than TDM (44). As a result, TDCM has been effectively used as an adjuvant in the Ribi Adjuvant system (45, 46). To date, however, only one study involving the use of a NFAT-GFP reporter assay has demonstrated that a representative TDCM (**2**) bound and activated hMincle and mMincle (47). Thus, while Nishizawa et al. synthesized a series of individual TDCMs and found that the immunoadjuvant activity of the TDCMs is associated with the (2*S*, 3*S*) and the (2*R*, 3*R*) diastereomers, and not the (2*R*, 3*S*) or (2*S*, 3*R*) diastereomers (42), and that the β-methoxy TDCM had similar activity to the β-hydroxy TDCM in a phagocytic assay (43), further studies are required in order to determine whether the immunomodulatory response to the aforementioned TDCMs is Mincle dependent.

### Other Branched TDEs

In addition to the α-branched TDMs **1** and TDCMs **2**, a number of α- (**3**), β- (**4**), γ- (**5**), and iso-branched (**6**) TDEs have been synthesized over the years and their immunomodulatory properties explored (Figure 3). Examples include a series of α-, β-, and γ-branched TDEs (**3**, **4**, and **5**), including Vizantin (**4b**), as first prepared by Yamamotoet al. (43), with Vizantin and functionalized probes (e.g., fluorescent, bead-conjugated) later being synthesized by Oda et al. (49). However, to date, none of these ligands have been explored for their ability to bind and activate Mincle. Vizantin, which contains two linear alkyl chains at the β-position of the ester, is considered to be an analog of TDM and TDCM (43), and therefore might be expected to bind and activate Mincle. Notwithstanding, Vizantin and its analogs have been found to bind to TLR-4. A simplified Vizantin monoester (*vide infra*) has been found to show weak activation of Mincle (50), further suggesting that Vizantin itself might be able to activate Mincle; however, this remains to be determined.

Iso-branched TDEs, also known as maradolipids, were first isolated in 2010 from the dauer larva stage of *Caenorhabditis elegans* (51) and synthesized shortly thereafter (52–54). Our group recently determined that maradolipids (**6a–f**, Figure 3) are Mincle-ligands (48). On the whole, the iso-branched maradolipids led to greater cytokine production (IL-6, IL-1β, IL-12, IL-12, MIP-2 by BMMs), and a faster immune response when compared to their linear counterparts. This is quite remarkable given the subtle structural difference between the linear and iso-branched TDEs. Moreover, only the iso-branched TDEs with a carbon chain of >C12 lead to a strong inflammatory response. This observation is in line with studies using the non-branched linear TDEs (*vide infra*).

### Linear TDEs

#### Straight Chain TDEs

**Figure 4:**
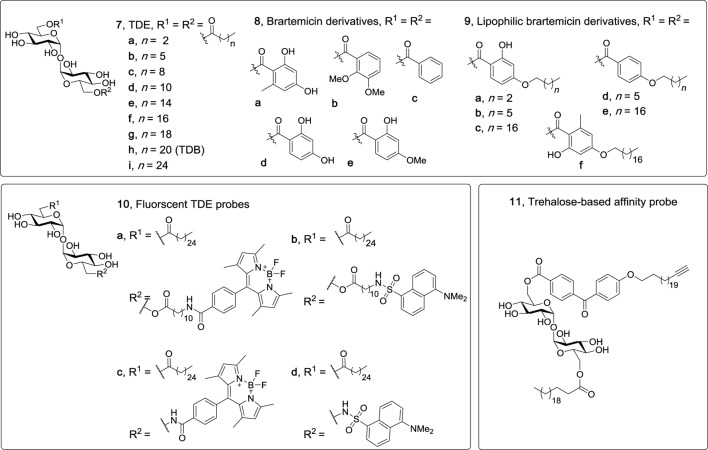
Non-branched trehalose diesters (TDEs), including linear TDEs (60), brartemicin derivatives (61, 62), fluorescent TDE probes (63), and a trehalose based affinity probe (64). Of these derivatives, only the linear TDEs (**7d–i**), the lipophilic brartemicin derivatives (**9a–f**), and the fluorescent TDE probes (**10a–d**) have been shown to be macrophage inducible C-type lectin agonists.

Trehalose dibehenate (TDB, **7h**, Figure 4), which contains behenic acid (C22) esterified to α,α′-trehalose, is one of the most simple and most studied Mincle ligands. As mentioned above, TDB was first identified as a Mincle ligand by Ishikawa et al. in 2009 (6), with contemporary studies by the Lang group illustrating that TDB engages the TLR-independent Syk/Card9-dependent pathway (7, 8). Although the receptor for TDB was only recently determined, there has been longstanding interest in this glycolipid with its first synthesis being described in 1978 (29), and with applications that include anti-tumor (55) and anti-bacterial activities (29, 56). Prior to the identification of TDB as a Mincle agonist, TDB was also incorporated into dimethyldioctadecylammonium (DDA) liposomes to generate the CAF01 adjuvant system which has been shown to improve T cell responses (57) and to induce protective immune responses in various disease models including influenza (58), tuberculosis, chlamydia, and malaria (59).

To explore the influence of lipid length on macrophage activation, our group synthesized a variety of linear TDEs (C4-C26, **7a–c,f–i**) and analyzed the ability of the diesters to activate BMMs, as determined by measuring NO production and the secretion of cytokines IL-6 and IL-1β (60). From this work, it was determined that a lipid length of >C12 was required to activate macrophages, with the greatest amount of IL-6, IL-1β, and NO being produced by the C22 (**7h**) and C26 (**7i**) TDEs. In subsequent studies, we confirmed that this immune response was Mincle-dependent, and also demonstrated that the C12-TDE (**7d**) elicited the production of MIP-2 by BMMs, but not other pro-inflammatory cytokines (IL-1β, IL-6, IL-12), thereby providing the first example of a TDE with a chain length of ≤ C12 leading to a Mincle-dependent immune response and one that is less inflammatory in nature (48). In related studies, Kallerup et al. demonstrated that the incorporation of the C16 (**7e**) and C18 (**7f**) TDEs into DDA liposomes led to a similar *in vivo* immunological response to that elicited by TDB (**7h**)/DDA liposomes (65). Thus, it appears as if the subtleties of the *in vitro* responses to TDEs may have less of an effect *in vivo* and/or that the presentation of the glycolipids can alter their immunomodulatory properties, with the immunomodulatory DDA potentially having a greater influence on the overall immune response. Notwithstanding, further *in vitro* and *in vivo* studies using other Mincle agonists are required to test these ideas.

#### Brartemicin Analogs

The natural product brartemicin is a TDE with two substituted benzoates esterified to the 6- and 6′-positions (**8a**, Figure 4). Brartemicin (**8a**) was recently isolated from the culture broth of an actinomycete of the genus *Nonomuraea* and the natural product, along with synthetic analogs (66), were found to have an inhibitory effect on cancer cell invasion (67). Given the structural resemblance of brartemicin to other trehalose-based Mincle ligands, there has been much interest in determining whether brartemicin and/or analogs can bind and activate Mincle. In 2015, Jacobsen et al. determined that brartemicin is a potential ligand for Mincle by performing a series of competitive binding studies (61). Here, it was revealed that brartemicin (**8a**) and its 2,3-dimethoxy analog (**8b**) bound Mincle with a high affinity of *K*_I_ = 5.5 ± 0.9 and 5.4 ± 0.3 µM, respectively. Removal of substituents on the benzene ring (**8c**) resulted in a relatively lower affinity of 11.3 ± 0.9 µM. In these studies, it was hypothesized that the aromatic interactions between the brartemicin derivatives and Phe197 and Phe198 in Mincle accounted for the improved binding affinity of brartemicin (**8a**). Furthermore, *epi-*(α,β′)-brartemicin was synthesized and tested but its ability to bind Mincle was completely abolished. Molecular docking of brartemicin in bMincle using the Glide algorithms further suggested that one of aromatic esters of brartemicin interacted with the hydrophobic grove (Leu172, Val173, Val194, Phe197, Phe198) adjacent to the Ca^2+^-ion, while the second aromatic ester was orientated in the opposite direction with potential π-cation interactions with Arg182. In 2016, Feinberg et al. obtained a crystal structure of brartemicin and its analog binding to the extended CRD of bovine Mincle (68). Notwithstanding, no functional assays with the aforementioned brartemicin analogs were undertaken in either of these studies.

In more recent studies by our group, we synthesized a variety lipophilic brartemicin analogs (e.g., **9a–f**), including the natural product itself (**8a**) and other non-lipidated derivatives (**8c**,**d**), with the idea that a lipophilic tail would enhance Mincle binding (62). Surprisingly, we demonstrated that brartemicin (**8a**) did not bind either human or murine Mincle, as determined *via* an enzyme-linked immunosorbent assay (ELISA) using soluble Mincle-Ig fusion-proteins, and moreover, that **8a** did not activate NFAT-GFP reporter cells expressing mMincle or hMincle, or cause BMMs to produce IL-6, IL-1β, TNF-α, or MIP-2. In contrast, the medium chain length (C7) C7-brartemicin analogs (**9b,d**) showed better binding to hMincle and mMincle when compared to the longer chain length (C18) analogs (**9c**,**e**,**f**), however, the C18 brartemicin derivatives led to a stronger functional immune response (as determined by activation of the mMincle and hMincle NFAT-GFP reporter cell lines and cytokine production by BMMs), with activity being abolished when using Mincle^−/−^ cells. This observation was intriguing and suggests that Mincle binding does not necessarily correlate to a functional immune response and that longer chain lipids are required for a robust immune response to trehalose-based Mincle ligands. Building on the computational studies of Jacobsen et al. (61), we then used site-directed mutagenesis to determine that Arg183 (in hMincle) was essential for the adjuvanticity of our lead brartemicin analog C18dMeBrar (**9c**). We subsequently explored the potential adjuvanticity of C18dMeBrar (**9c**), including *in vivo* immunization studies using the model antigen ovalbumin, and demonstrated that **9c** leads to excellent Th1 adjuvant activity better than that of TDB (**7h**).

#### TDE Probes

Functionalized TDE molecular probes have also been found to bind and activate Mincle, thereby demonstrating the capacity of Mincle to accommodate larger functional groups. In the first of such studies, we demonstrated that BODIPY- and dansyl-functionalized TDEs, **10a,c** and **10b,d** (Figure 4), respectively, activated macrophages in a Mincle-dependent manner (63). In earlier studies, we synthesized an affinity-based benzophenone and alkyne functionalized trehalose probe (**11**) as a tool to study TDB/protein interactions, and determined that probe **11** was able to activate BMMs (as measured by NO production), albeit to a lesser extent than TDB (64). While the activity of probe **11** was not assessed using Mincle^−/−^ BMMs, it would seem likely that **11** bound Mincle and that the reduced activity of **11** compared to TDB might be due to the presence of the bulky benzophenone group near Mincle’s EPN motif. This suggests that while the Mincle binding site can accommodate a variety of ligands, the positioning or type of electrophilic trap (in this case the benzophenone group) might need optimization in order to generate a more active affinity-based trehalose glycolipid probe.

## Trehalose Monoesters (TMEs)

### Linear TMEs, Trehalose Monomycolates (TMMs), and Trehalose Monocorynomycolates (TMCMs)

**Figure 5:**
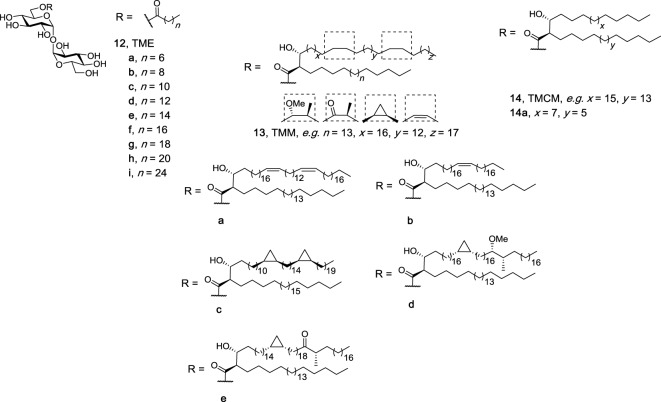
Structures of the trehalose monoesters (69, 71), trehalose monomycolates (27, 28), and trehalose monocorynomycolates (47), of which representative family members (e.g., **12h**, **12i**, **13d**, and **14a**) have been determined to be macrophage inducible C-type lectin agonists.

Several groups have investigated whether two lipophilic moieties attached to trehalose are required for effective Mincle binding and activation. An attraction of the use of monoesters of trehalose, as compared to their diester counterparts, is that monoesters can be comparatively easy to synthesize and may also have improved water solubility. Here, it is proposed that one lipid on the trehalose sugar binds to the major lipophilic groove in Mincle. In seminal work, Furukawa et al. performed surface plasmon resonance (SPR) binding assays using a set of TMEs with a single acyl chain with different lengths [C8 (**12a**), C10 (**12b**), C12 (**12c**)] (Figure 5) and demonstrated that a minimum lipid length of C10 was required for lipid binding (18). No functional immune response to the TMEs, however, was determined. In the first study to demonstrate that TMEs could activate Mincle, our group synthesized the C22 (**12h**) and C26 (**12i**) TMEs and demonstrated that solubilized C22 and C26 TMEs led to the production of NO and IL-6 by BMMs in a Mincle-dependent manner (69). In our studies, NO and IL-6 production by BMMs in response to the C22 and C26 TMEs was comparable to that of the C22 TDB, thereby suggesting that only one lipid is sufficient to lead to robust Mincle binding and activation. Since these studies, naturally occurring TMEs have been identified in the lipid extracts of *C. elegans* larvae at the dauer and L3 stages, albeit to a lesser extent than TDEs (70).

In 2016, Huber et al. reported that TDEs were more potent than their corresponding TMEs when assessing the activity of the compounds using murine BMMs in plate-coating assays (71). Using a variety of TMEs containing different lipid lengths [C14 (**12d**), C16 (**12e**), C18 (**12f**), C20 (**12g**), C22 (**12h**)], the authors observed a significant decrease in both G-CSF and NO production when comparing TMEs to their corresponding TDEs. Notwithstanding, the differences between Lang’s studies and ours could be explained by the subtleties in the types of assays used. Indeed, during the assessment of related TMEs (e.g., TMMs, TMCMs, *vide infra*), no significant difference between the monoesters and diesters was observed.

Several more elaborate TMEs, the TMMs, and the TMCMs, have also been tested for their ability to bind and activate Mincle. TMMs have been isolated from the wax D fraction of virulent human *M. tuberculosis* as mixtures of compounds (25); however, to better understand how functionalization along the mycolic acid group influences the immunomodulatory property of the compounds, 13 TMMs were synthesized, with 11 subsequently being tested for their immunomodulatory properties, including **13a**–**13e** (Figure 5) (27). All synthesized TMMs led to cytokine production by BMDCs in a plate-bound assay, with a dose–response analysis being performed with nine pairs of TMMs and TDMs composed of the same mycolate moiety. Here, the number of mycolate chains bound to trehalose influenced TNF-α and IL-6 production by BMDCs *in vitro*, with a lower cytokine response being observed following stimulation with the TMMs. This trend, however, was not seen in *in vivo* with representative TMM **13d** exhibiting similar immunostimulating potential as both TDM and TDB in an OVA immunization model. The immune response to TMM **13d** was determined to be Mincle dependent. When van der Peet et al. tested their synthesized TMCM (**14a**) using NFAT-GFP reporter cells expressing hMincle and mMincle, TMCM was found to be just as active as its diester counterpart (47). Taken together, these results suggest that a second lipid on the trehalose moiety might not be necessary to make an effective adjuvant and that subtle differences between the different immunomodulatory compounds might be observed when different immunological assays are used.

## Monosaccharide Esters

### Glucose Monoesters

**Figure 6:**
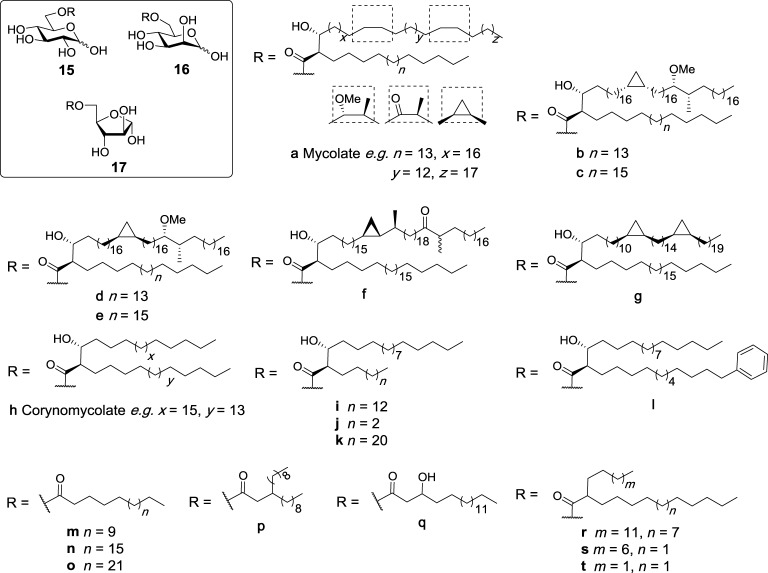
Glucose monoesters have been isolated from different species of *Mycobacterium* and *Corynebacterium*, including glucose monomycolates (**15a–g**) and glucose monocorynomycolates (**15h–l**). These, and related monosaccharide monoesters (**15m–r**) have been examined for their ability to activate macrophage inducible C-type lectin (Mincle), with specific glucose monoesters (e.g., **15a**,**b**,**i**–**l**,**n**,**p**–**t**, **16a**,**h**,**r**, and **17b**) being selected and found to directly bind and activate Mincle (27, 47, 50, 75–77).

Glucose monomycolate (GMM, **15a**, Figure 6) is an antigenic glycolipid that has been isolated from different species of corynebacteria and mycobacteria including *M. tuberculosis* (72, 73), and was initially found to be presented to antigen-specific T cells by CD1b in humans (74). GMM can be generated by trehalase treatment of TDM, and GMM generated in this manner was initially not found to be a Mincle ligand (6). In contrast, more recent studies concluded that GMM is indeed a Mincle ligand (27, 47), whereby the lack of Mincle dependency in the first set of Mincle assays was attributed to the presence of esterases in the enzyme mixture, which may have cleaved the lipids (47).

Several syntheses of GMMs have been reported, with the first partially synthetic GMM being prepared by Prandi in 2012 in good overall yield (26%) *via* the esterification of benzyl 2,3,4-tri-*O*-benzyl-6-*O*-tosyl-β-d-glucopyranoside with a mixture of mycolic acids purified from *M. tuberculosis* (78). A series of distinct and enantiopure mycolic acids have also been used to prepare several homogeneous GMMs (**15b–h**, Figure 6) (75). Subsequent biological evaluation revealed that GMMs with a *cis*-methoxy mycolic acid (**15b**) induced production of TNF-α, IL-6, IL-12p40, and IL-1β from BMDCs in a dose-dependent manner, albeit with lower potency than TDB (27). Cytokine production was found to be Mincle dependent as activity was completely abolished in Mincle^−/−^, FcRγ^−/−^, and Malt1^−/−^ mice. These observations were further confirmed using HEK-Mincle reporter cells, where GMM **15b** showed comparable activity to TDB. GMMs containing different mycolates (**15d–g**) were also able to stimulate BMMs to produce TNF-α at levels similar to that achieved *via* the stimulation of BMMs with TDB, however, no significant difference was observed between the different classes of mycolic acids. Further studies by the same authors revealed that synthetic GMM **15b** activated the NLRP3 inflammasome through the same mechanism as TDB, with the *in vivo* adjuvanticity of GMM (as determined by an OVA immunization model) demonstrating that GMM exhibited lower immunostimulatory potential than both TDM and TDB. Notwithstanding, the ability of GMM to activate both the innate and adaptive immune systems makes it an interesting adjuvant for further vaccination studies.

Glucose monocorynomycolate (GMCM, **15h**, Figure 6) is a simplified analog of TDCM previously isolated from *Corynebacterium glutamicum* when glucose was used as the carbon source (79). Recently, van der Peet et al. described the first synthesis of (+)-corynomycolic acid (CMA) through a previously reported boron mediated aldol reaction which allows for the generation of *anti*-configured lipids (47). The synthetic CMA was then coupled to benzyl 2,3,4-tri-*O*-benzyl-6-*O*-tosyl-β-d-glucopyranoside and deprotected to generate enantiopure GMCM **15i** in excellent yield. The synthetic GMCM was then compared to TDM, TDCM, TMCM, and glucose monobehenate (GMB, **15n**, *vide infra*) for Mincle activation using the NFAT-GFP reporter cell assay and was found to activate both human and mouse Mincle, albeit with lower potency than TDM, TDCM, and TMCM. Although GMCM (**15i**) was able to activate the reporter cell lines, GMB (**15n**) was inactive in this assay, highlighting the requirement of substituents in the α- and β-positions of the lipid chain in glucose monoesters for strong Mincle activation. Direct binding of the active ligands to Mincle was confirmed using the human and mouse Mincle Ig fusion protein assay. The same group later synthesized derivatives of CMA in order to investigate the effects of changes in the lipid portion on Mincle activity (50). The synthetic GMCM analogs contained a shorter α-pentyl chain (**15j**), a longer α-tricosyl chain (**15k**), or a more complex α-phenyldodecyl chain (**15l**). A truncated version of Vizantin, which consists of glucose esterified to a β-branched achiral lipid (**15p**), was also synthesized. All four analogs were assessed for their Mincle activity and compared to TDM, TDB, GMCM and GMB using the NFAT-GFP reporter cell assay. Here, TDM, TDB, and GMCM (**15i**) signaled through both mouse and human Mincle with similar potencies, and surprisingly, GMB now showed weak signaling, albeit only at the highest concentration tested (1 nmol). The shorter α-branched chain GMCM analog **15j** and the simplified Vizantin analog **15p** also only showed notable Mincle activity at the highest concentration tested. Notwithstanding, the longer α-branched GMCM analog **15k** showed dose-dependent Mincle activity similar to GMCM **15i**, emphasizing the requirement of a longer α-branched chain for potent Mincle activity. The modified lipid analog **15l** also showed similar activity to GMCM **15i**.

Decout et al. recently synthesized simplified glucose C16 (**15m**), C22 (GMB, **15n**), and C28 (**15o**) monoesters (Figure 6) and compared their ability to bind and activate Mincle relative to synthetic TDM, TDB, and glucose monoesters with 3-hydroxyoctadecanoic acid (Glc3OHC18, **15q**) or 2-tetradecyloctadecanoic acid (GlcC14C18, **15r**) (77). HEK-Mincle reporter cells were used for assessing Mincle activity, where HEK cells expressing either human or mouse Mincle were coupled to an NF-κB–inducible reporter system using alkaline phosphatase. In comparison to the NFAT-GFP assay where GMB showed minimal activity (50), GMB showed no Mincle activity with HEK-Mincle cells and this lack of activity was also observed for the C16 and C28 monoesters. GlcC14C18 (**15r**), however, showed potent activity for both human and mouse Mincle similar to that seen for TDM, while Glc3OHC18 (**15q**) was unable to activate Mincle (77). Glc3OHC18 (**15q**) was also unable to bind a soluble form of hMincle, whereas GlcC14C18 (**15r**) showed direct binding, confirming the observed activity. GlcC14C18 (**15r**) was also able to induce the Mincle dependent production of pro-inflammatory cytokines TNF and IL-6 effectively by mouse BMMs and BMDCs and human monocyte-derived macrophages. The adjuvant properties of **15r** were then investigated *in vivo*, whereby incorporation of **15r** into DDA liposomes resulted in a significant increase in IL-2, IFN-γ, and IL-17 compared to TDB, whereby the latter did not cause a significant increase in cytokine levels. Moreover, **15r** was found to be less toxic than TDB, and also induced protective immunity in a mouse model of *M. tuberculosis* infection. Altogether, these results suggest that GlcC14C18 (**15r**) represents a potential new class of simple α-branched glycolipid Mincle ligands with promising adjuvant properties.

To understand more about the ability of the glucose monoesters to bind and activate Mincle, Decout et al. preformed a series of molecular dynamic studies with Glc3OHC18 (**15q**) and GMCM (**15h**) (77). Using the crystal structure of bovine Mincle, the authors demonstrated that the α-alkyl chain of **15h** bound to a short hydrophobic groove, which resulted in widening of the main hydrophobic groove. This new conformation better accommodated the main alkyl chain inside the cleft. In contrast, Glc3OHC18 (**15q**) lacked this interaction, thereby causing the lipid chain to sit on the surface instead of inside the hydrophobic groove. These studies suggested that the presence of a lipid containing an α-branch of at least four carbons at the 6-position of glucose is a requirement for Mincle signaling. To confirm this finding, synthetic GlcC9C12 (**15s**) and GlcC4C12 (**15t**) were evaluated for their ability to bind mMincle and hMincle. Both of the shorter analogs **15s** and **15t** were Mincle agonists, with EC_50_ values comparable to GlcC14C18 (**15r**) for mMincle and lower activity in hMincle. Next, mutagenesis studies were undertaken where the Mincle A174 residue that is involved in lipid recognition was changed to phenylalanine. This weakly improved the binding of **15r** and TDB, however, when the residue was changed to proline, the binding was decreased for **15r**. Taken together, these results suggest that the introduction of an aromatic side chain at position 174 in Mincle can slightly improve binding of the lipid in the main hydrophobic groove, without significantly interfering with binding of the 2-alkyl chain in the short hydrophobic groove.

### Mannose Monoesters

As C-type lectins, such as Mincle, typically bind mannose and glucose residues (2), it has been proposed that Mincle may be able to bind mannose-derived glycolipids. To this end, Decout et al. synthesized mannose monomycolate (ManMM, **16a**, Figure 6), mannose monocorynomycolate (ManMCM, **16h**) and mannose 2-tetradecyloctadecanoate (ManC14C18, **16r**) (Figure 6) and assessed these derivatives for their ability to activate Mincle using HEK-Mincle reporter cells (77). All three compounds bound soluble hMincle and activated both mMincle and hMincle reporter cells, albeit with lower potency than their glucose counterparts (described above). Similar to GlcC14C18 (**15r**), ManC14C18 (**16r**) was also able to induce Mincle dependent pro-inflammatory cytokine production from BMMs and BMDCs and human monocyte-derived macrophages (moMφs). The incorporation of ManC14C18 (**16r**) into DDA liposomes also resulted in the production of IL-2, IFN-γ, and IL-17 *in vivo*; however, the result was deemed statistically insignificant and so no further testing was carried out with this ligand.

### Arabinose Monoesters

Mohammed et al. reported the synthesis of six arabinose monomycolates from single synthetic mycolic acids (76), with several of these substrates being subsequently tested for their ability to induce cytokine production by BMDCs (27). Here, AraMM with a *cis*-methoxy mycolic acid (**17b**, Figure 6) induced the production of pro-inflammatory cytokines TNF-α, IL-6, IL-12p40, and IL-1β; however, cytokine production was lower for **17b** than that induced by TDB and *cis*-methoxy GMM **15b**. AraMMs containing different mycolic acids (**17c,f**) led to comparable levels of TNF-α production by BMMs to that induced by **17b**.

To explore the mode of action of the AraMMs, AraMM **17b** was used as a representative substrate, with TNF-α production through stimulation with **17b** being found to be Mincle dependent and MyD88 independent (27). This finding was contradictory to a previous study where natural arabinose mycolates from *M. bovis* were reported to induce TNF-α through the MyD88 and TLR2 pathways (80). However, the natural arabinose mycolates consisted of a mixture of mono-arabinose mono-mycolates, tetra-arabinose tetra-mycolates, penta-arabinose tetra-mycolates, and hexa-arabinose tetra-mycolate; therefore, it is possible that some of these glycolipids signal through MyD88. The Mincle activity of **17b** was further confirmed by its ability to activate HEK-Mincle reporter cells, albeit only at a concentration 100-fold higher than that of TDB. AraMM **17b** also induced the production of IL-1β through NLRP3-dependent mechanisms similar to GMM and TDB, however, *in vivo* studies revealed that **17b** did not induce local inflammation and did not have adjuvant properties (27). Although the adjuvanticity of AraMM **17b** was modest compared to the other mycolic esters in this study, this work nonetheless represents the first report of Mincle being activated by pentose-derived glycolipids.

## Glycerides

### Mono-Acyl Glycerides

**Figure 7:**
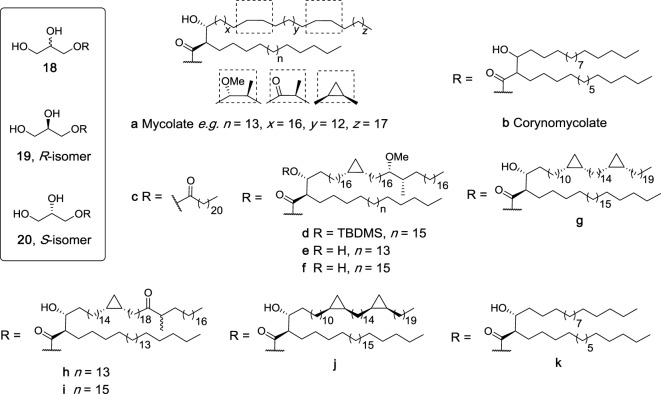
Structures of synthetic glycerol monoesters that have been evaluated as macrophage inducible C-type lectin (Mincle) ligands (27, 47, 84, 85). Of these, **18a**, **18b**, **18c**, **19k**, and **20k** have been determined to be agonists for human Mincle and not murine Mincle.

Glycerol monomycolate (GroMM, **18a**, Figure 7) is a mycobacterial lipid that is perhaps best known for its ability to be presented to T cells by CD1b (81). In recent years, there has been much interest in the potential of GroMM (**18a**) and the simpler synthetic analog glycerol monocorynomycolate (GroMCM, **18b**) as vaccine adjuvants, with both glycolipids having *in vivo* adjuvanticity comparable to TDB when incorporated into DDA liposomes (82, 83). However, it was only in more recent studies that isolated GroMM (**18a**) was found to be capable of activating hMincle reporter cells in a dose-dependent manner similar to TDM, albeit with less potency (84). In contrast, this activity was absent in mMincle reporter cells, which recognized TDM. Similarly, synthetic glycerol monobehenate (GroMB, **18c**) was able to activate human but not mouse Mincle (84).

To better understand this species-specific activity, extracellular domain swaps between mouse and human Mincle were undertaken, leading to the conclusion that the hMincle ectodomain may be involved in GroMM recognition (84). Furthermore, site-directed mutagenesis studies were performed where the amino acid residues at positions 174–176 or 195–196 (or both) in mMincle were swapped with those of hMincle. The single mouse mutants showed activity with GroMM (**18a**), while the doubly mutated mouse Mincle showed no activity with either TDM or GroMM (**18a**) due to major conformational changes in the protein. *In vitro* assays revealed that **18a** was capable of inducing TNF-α production from hMincle transgenic mouse derived macrophages, but no induction was seen with non-transgenic mouse derived macrophages. This response was also confirmed *in vivo*, where injection of GroMM liposomes caused a local inflammatory response only in hMincle transgenic mice. Finally, TNF-α production induced by **18a** was completely abolished in the presence of an antibody against hMincle. Altogether, this study confirmed the first differential recognition of GroMM (**18a**) as ligand for human but not mouse Mincle.

The synthesis of four mycolate esters of *R*-glycerol (**19d**,**e**,**g**,**h**, Figure 7) and five esters of *S*-glycerol (**20e–g**,**i**,**j**) have been described very recently and based on their structural similarity to the known glycerol-containing Mincle agonists, these mycolate esters of glycerol were suggested to be Mincle ligands (85). In these studies, however, *R*-GroMMs **19d**,**e**,**g**,**h** were unable to induce cytokine production by BMDCs, while no results for the *S*-isomers were reported. Notwithstanding, it has been suggested that such mycolate esters may play a key role in the stimulation of CD1b-restricted germline-encoded, mycolyl-reactive (GEM) T cells, a process that can also be modulated by the corresponding free mycolic acids (85).

In 2015, the enantiopure CMA esters of *R*- (**19k**) and *S*-glycerol (**20k**) were synthesized by van der Peet et al. and subsequently found to activate hMincle NFAT-GFP reporter cells in a dose-dependent manner similar to TDM (47). No significant activity was observed for reporter cells bearing mMincle. This result was in agreement with the previously described study by Hattori et al. where GroMM (**18a**) only signaled through human but not mouse Mincle (84). Moreover, the *S*-isomer **20k** had significantly higher activity for hMincle compared to the *R*-isomer. This was the first instance where the ability of Mincle to differentiate between isomers was demonstrated.

### Glycosyl Glycerides

**Figure 8:**
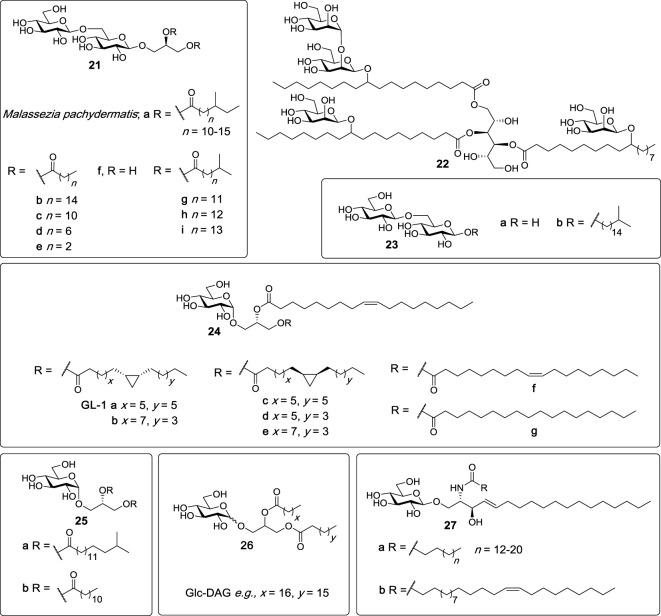
Several glycosyl diacylglycerides have been isolated from bacterial species such as the *Mycobacterium tuberculosis, Streptococcus pneumoniae*, and *Lactobacillus plantarum*, as well as rom *Malassezia* sp. and shown to be macrophage inducible C-type lectin (Mincle) ligands (86–90). Mincle agonists include glyceroglycolipids (e.g., **21a**–**c**, **g**–**i**), mannoside (**22**), and glucosyl diglycerides (**24a**–**g**, **25a**,**b**, and **26**). β-Glucosylceramides (**27a**,**b**), isolated from dead human and mouse cells, have also been determined to be Mincle agonists (91).

In 2009, Yamasaki et al. analyzed more than 45 species of pathogenic fungi using NFAT-GFP reporter cells expressing FcRγ with Mincle in search for exogenous Mincle ligands (5). Later studies found that Mincle only selectively recognized *Malassezia* species among all the species tested, with the Mincle ligands being subsequently identified (86). The ligands consist of glyceroglycolipids (**21a**, Figure 8) which are structurally similar to lipoteichoic acid (LTA) anchors, and which contain one glycerol and one gentiobiose (6-*O*-β-d-glucopyranosyl-glucopyranose). LTA, however, does not possess Mincle activity. In addition, a more complex Mincle ligand, compound **22**, which has a mannitol backbone and which is esterified to two 10-*O*-β-d-mannopyranosyl-10-hydroxy-octadecanoic acids and one 10-*O*-(β-d-mannopyranosyl-[1→2]-β-d-mannopyranosyl)-10-hydroxy-octadecanoic acid, was identified. Both **21a** and **22** had Mincle agonist activity almost as potent as TDM, as determined using the hMincle/mMincle NFAT-GFP reporter assay, and direct Mincle binding was confirmed using soluble Mincle-immunoglobulin (Ig) protein ELISAs. BMDCs were able to secrete TNF in response to **21a** and **22** in a Mincle-dependent manner.

In 2015, Richardson et al. synthesized a series of β-gentiobiosyl diacylglycerides (87), which were previously isolated from *M. tuberculosis* H37Ra (88), as well as additional β-gentiobiosyl analogs. The activity of synthetic *M. tuberculosis* β-gentiobiosyl diglycerides (**21b**,**g–i**, Figure 8) was then compared with TDM in an NFAT-GFP assay, whereby it was determined that all four compounds signaled only through mMincle, albeit weakly compared to TDM. Next, β-gentiobiosyl diacylglycerides with acyl chain lengths of C12 (**21c**), C8 (**21d**), and C4 (**21e**), gentiobiosyl glycerol (**21f**), free gentiobiose (**23a**), and iso-C17 gentiobioside (**23b**) were assayed using the Mincle NFAT-GFP reporter assay. Here, it was determined that only the C12 analog **21c** was able to activate mMincle, albeit less so than TDM, with the same ligand also leading to the weak activation of hMincle. Evidence for direct binding was obtained by a solubilized Mincle Ig fusion assay, where **21c** bound mMincle but not hMincle. To obtain a functional read-out, compounds **21b-e** were also tested for their ability to activate BMDCs through production of TNF and MIP-2. Consistent with the NFAT-GFP assay, significant cytokine production was only observed with **21c**. The authors proposed that the disaccharide moieties of the gentiobiosyl diglycerides bind to the Mincle CRD in the same manner as trehalose, with the first glucose residue engaging the Ca^2+^ in the primary site, and with the acyl chains binding to the hydrophobic groove adjacent to the sugar binding site.

Other glycosyl glycerides include the glucosyl diglycerides, which are a class of compounds first isolated in the mid 1960s from *Lactobacillus plantarum—*a bacterium that is part of the microbiota in the human mucosa (92). However, it was only in 2012 that our group structurally characterized GL-1 (**24a**) as the major glycolipid present in this bacterium (93). Subsequently, GL-1 (**24a**), and six analogs (**24b–g**) consisting of different lipids on the *sn*-1 glycerol position, were synthesized in seven steps from allyl α-d-glucopyranoside by Shah et al. and compared to TDM and TDB for Mincle activation using NFAT-GFP assays (89). Compounds **24a–g** showed almost identical dose-dependent Mincle activity for both mouse and human Mincle, however, this activity was lower than TDM and TDB. These results suggested that both fatty acid chains do not require a cyclopropane ring or an alkene to signal through Mincle.

Richardson et al. also explored the Mincle-binding capacity of iso-C_17_ glucosyl diglyceride (**25a**), with these studies being undertaken to probe the effect of the loss of the gentiobiosyl moiety on Mincle activation (87). Here, **25a** displayed superior signaling through both mouse and human Mincle compared to all the other gentiobiosyl analogs, including the most active C12 analog (**21c**), as determined using the NFAT-GFP reporter assay. The ability of **25a** to activate cells was also confirmed *via* the production of TNF and MIP-2 upon the stimulation of BMDCs with **25a**. Consequently, the glucosyl diglyceride with a C12 lipid (**25b**) was synthesized; however, this new analog showed reduced mMincle activity and no hMincle activity compared to its iso-C17 (**25a**) counterpart. These results suggest that iso-branched lipids are potentially required for more robust human but not mouse Mincle signaling and that only one glucose moiety is sufficient to achieve significant Mincle binding and activity.

In 2016, Behler-Janbeck et al. reported that Mincle expression peaked after lung infection with *Streptococcus pneumoniae*, with subsequent analysis revealing that glucosyl diacylglycerol (Glc-DAG, **26**) (Figure 8) was the glycolipid in *S. pneumoniae* that triggered Mincle reporter cell activation (90). Here, the identity of the natural ligand was confirmed by comparison to synthetic Glc-DAG. Studies revealed that Glc-DAG (**26**) was able to induce TNF-α and MIP-2 production by bone-marrow-derived phagocytes in a Mincle-dependent manner. A significantly higher mortality rate was observed in Mincle^−/−^ mice compared to wild type (WT) mice in the focal pneumonia model (caused by *S. pneumoniae*) and not invasive pneumonia. Moreover, the antibacterial and dysregulated cytokine responses in mice challenged with focal pneumonia were normalized in Mincle^−/−^ mice that were reconstituted with a WT hematopoietic system. Collectively, these results indicate that Mincle plays a crucial role in lung protective immunity, specifically in focal pneumonia caused by *S. pneumoniae*.

## Monosaccharides for Mincle and MCL Binding

Lee et al. investigated the binding specificities of many CLRs, including Mincle and macrophage C-type lectin (MCL), against different glycans that were tested as BSA-conjugated neoglycoproteins (94). Both Mincle and MCL had the highest binding affinity to mannose and fucose, with lower affinities for *N*-acetyl glucosamine (GlcNAc) and glucose (Glc). Interestingly, Mincle had higher binding for galactose (Gal) and *N*-acetyl galactosamine (GalNAc) than MCL. Mincle was able to bind Lewis^x^-BSA, but MCL lacked this binding capacity. Moreover, both CTLs showed binding of lactose and *N*-acetyl lactosamine, but the binding affinity was less than Gal. Similarly, Glc disaccharides were bound less effectively than Glc, with the exception of MCL, which bound maltose better than Glc. The ability of the glycans to activate Mincle, however, was not determined.

## β-Glucosylceramide

In studies aimed at identifying endogenous Mincle ligands associated with cell death, Nagata et al. fractionated and analyzed the supernatant of dead cells using Mincle NFAT-GFP reporter cells (91). Subsequent mass spectrometry and NMR analysis revealed that the most active cell isolate fraction contained a mixture of six β-glucosylceramides (β-GlcCer) (**27a,b**); C16:0, C18:0, C20:0, C22:0, C24:0, and C24:1 (15Z) (Figure 8). When compared to TDM, synthetic β-GlcCer (C24:1) (**27b**) was able to activate NF-κB reporter cells as well as NFAT-GFP reporter cells expressing either mMincle or hMincle, with the importance of the glucose moiety being highlighted since ceramide, lactosyl ceramide and galactosyl ceramide did not act as Mincle ligands. β-GlcCer did not activate MCL, Dectin-2, or DCAR. β-GlcCer (24:1) (**27b**) was also capable of inducing TNF and MIP-2 production through the stimulation of BMDCs and this induction was absent in Mincle^−/−^ BMDCs. Moreover, cytokine production was dependent on Card9 activation and did not require TLR signaling through the MyD88 pathway. Altogether, these results confirm that β-GlcCer activates myeloid cells in a Mincle-dependent manner and is the first example of a carbohydrate-derived endogenous ligand being able to act as a potential adjuvant through Mincle-dependent mechanisms. Further studies indicated that β-glucosylceramidase (GBA1)-deficient mice had increased inflammation caused by cell death, but inflammation was lower in GBA1 and Mincle double-deficient mice suggesting that β-GlcCer induced inflammation may involve Mincle.

## Steroids

**Figure 9:**
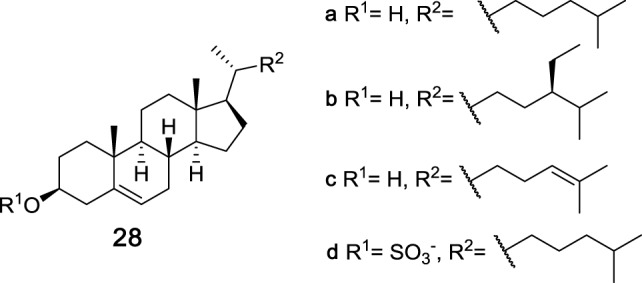
Structures of steroid ligands that bind macrophage inducible C-type lectin through an alternative binding site containing a cholesterol recognition/interaction amino acid consensus-like motif (20, 95).

While carbohydrates are the most commonly recognized class of Mincle ligands, steroids have also been found to bind Mincle and induce a proinflammatory response. Cholesterol (**28a**, Figure 9) has been known to have inflammatory properties for some time; however, the first innate immune receptor for cholesterol, hMincle, was only identified in 2015 by Kiyotake et al. (20). Here, hMincle was identified as the receptor for plate-coated and crystalline cholesterol using a NFAT-GFP reporter assay, while NFAT-GFP reporter assays involving mMincle and rat Mincle (rMincle) were not activated by cholesterol. As part of their studies, Kiyotake et al. tested different cholesterol-based steroids and found that changes to the hydroxyl or alkene functionality or removal of the lipophilic alkyl chain abolished hMincle binding. However, hMincle tolerates slight changes in the lipophilic alkyl chain, with sitosterol (**28b**) and desmosterol (**28c**) also being hMincle agonists. Using RAW-Blue cells transfected with hMincle, it was determined that cholesterol crystals induce TNF and MIP-2, albeit at lower levels than TDM, with subsequent assays revealing that plate-coated cholesterol and cholesterol crystals upregulate inflammatory chemokines and cytokines in BMDCs.

To better understand how cholesterol might activate Mincle, Kiyotake et al. drew insight from previous studies that reported that cholesterol can interact with proteins through their cholesterol recognition amino acid consensus (CRAC)-like motif (96). This sequence of amino acids is found in hMincle but not murine Mincle. To determine whether cholesterol binds the CRAC-like motif in Mincle, Kiyotake et al. thus created a NFAT-GFP reporter cell line with a mutation in the CRAC-like motif of hMincle (20). The mutant hMincle lost its ability to recognize cholesterol crystals but retained its capability to recognize TDM in comparable levels to that of WT Mincle, thereby demonstrating that cholesterol and TDM bind to different sites in hMincle.

The ability of cholesterol sulfate (**28d**) to act as a Mincle ligand was recently determined by Kostarnoy et al. in studies aimed at identifying the role of Mincle in skin allergies (95). In this work it was determined that Mincle is upregulated after skin tissue damage in mice, with the resulting proinflammatory cytokine response being Mincle dependent. Using a HEK293–mMincle–NF-κB–Luc reporter cell line, the endogenous Mincle ligand was then found to be cholesterol sulfate (**28d**), which exhibited activity comparable to that of TDB in the reporter cell line. Compound **28d** was also able to induce the secretion of pro-inflammatory cytokines IL-1α and IL-1β, KC, and MIP-1α and MIP-1β and this induction was abolished in Mincle^−/−^ BMDCs. *In vivo* experiments revealed that injections with **28d** caused Mincle-mediated induction of local inflammation, with a contact hypersensitivity model subsequently being used to demonstrate that Mincle deficiency leads to a significantly lower inflammatory response and strongly suppressed clinical symptoms of allergy, suggesting that Mincle plays an important role in promoting skin allergies. The binding site for cholesterol sulfate (**28d**) in mMincle, however, was not determined in this study.

## Summary and Conclusion

Macrophage inducible C-type lectin is able to accommodate a variety of ligands, however to date, most studies have focused on carbohydrate-derived ligands that bind to Mincle’s CRD, which contains an EPN motif. As the major hydrophobic groove in Mincle is rather shallow and open-sided (18, 19), it is perhaps not surprising that Mincle can accommodate ligands that include both branched and linear chain glycolipids and other derivatives with bulkier (e.g., aromatic) functional groups. The presence of a second minor hydrophobic groove next to the EPN motif is also thought to be able to accommodate a second lipid, however, as illustrated by numerous examples (e.g., TMEs, glucose, and mannose monoesters), one lipid is often sufficient to lead to Mincle binding and activation. Mincle is also able to accommodate glycolipids with both α- and β-anomeric configurations.

When exploring the immunostimulatory properties of glycolipid Mincle ligands, subtle changes to functional groups along the lipid backbone have been found to affect the immune response. For example, studies with TDMs revealed slight differences in the ability of TDMs with different mycolic acids to activate BMDCs *in vitro*, whereby it was demonstrated that increased polarity in the middle of the meromycolate branch does not increase Mincle agonist activity (27). Similarly, iso-TDEs (maradolipids) led to greater cytokine production by BMMs *in vitro* compared to their linear counterparts (48). The ability of Mincle to differentiate between isomers has also been demonstrated in studies with optically pure CMA esters of *R*- and *S*-glycerol, whereby the *S*-isomers was better able to activate reporter cell lines expressing hMincle (47). In other instances, however, changes in functional groups along the lipid backbone did not lead to differences in the adjuvanticity of Mincle ligands, as illustrated in studies with GMMs and with glucosyl glycerides (27, 89).

More marked differences in the immunomodulatory potential of similar classes of Mincle agonists have been observed when changes were made to the length of the lipid chains. This was first demonstrated in studies with linear TDEs, with longer lipids leading to a stronger pro-inflammatory response *in vitro* (48, 60). A similar trend was observed with lipidated brartemicin derivatives, whereby it was also demonstrated that π-cation interactions were essential for the activity of the lead compound (62). The effect of lipid length on the immunomodulatory potential of Mincle agonists other than trehalose derivatives has also been demonstrated with, for example, longer chain GMCM and β-gentiobiosyl diacylglycerides derivatives showing enhanced Mincle activity compared to their shorter-chain counterparts (50, 87).

With regard to the glucose monoester and glucosyl glyceride Mincle agonists, lipid branching influences activity. It has been suggested that the presence of an α-branched lipid containing at least four carbons at the 6-position of glucose is required for Mincle activation (47). Moreover, iso-C17 gentiobioside, which contains only one lipophilic chain, was unable to activate Mincle, yet a similar derivative (gentiobiosyl diglyceride) with two C12 lipid chains was a Mincle agonist (87). In the case of the glucosyl diglycerides it has been suggested that iso-branched lipids are required for more robust human but not mouse Mincle signaling (87). In contrast, certain non-glycosylated Mincle agonists containing no lipid branching (e.g., GroMB) can activate Mincle, albeit only the human isoform (84). These results highlight the complexity and challenges of predicting which compounds might be the most potent Mincle agonists and illustrate the need to assess the immunomodulatory potential of each ligand class. Notwithstanding, lipid length and configuration appear to be important considerations when designing Mincle agonists.

There has also been interest in assessing the relative immunostimulatory properties between different classes of Mincle agonist. Such analysis can only be undertaken when the different classes of Mincle ligands are compared in the same assay, and thus, a comparative study of the different Mincle ligands to date is lacking. Notwithstanding, a number of studies offer some clues in this direction. For example, glucose esters show greater immunomodulatory potential than arabinose esters (27), with an earlier study demonstrating that glucose esters showed greater adjuvant capacity compared to analogous monoesters, with one GMCM (GlcC14C18) having better adjuvant activity in an OVA immunization model when compared to TDB (77). In another study, however, a glucosyl monomycolate derivate showed similar adjuvant activity to TDB in an *in vivo* OVA immunization model (27). Lipidated brartemicin derivatives have also shown enhanced *in vivo* adjuvant activity compared to TDB (62). In studies that identified the first endogenous ligand for Mincle, β-GlcCer, it was also demonstrated that ceramide, lactosyl ceramide and galactosyl ceramide were not Mincle agonists (91).

The most comprehensive studies aimed at comparing the relative immunostimulatory properties of Mincle agonists, however, relate to comparative merits of TMEs and their diester counterparts. Needless to say, the overarching conclusions from such studies are not always clear. *In vitro*, some studies have demonstrated that linear non-branched TDEs are more immunostimulatory than their diester counterparts (71), while other studies point to no significant difference between the two classes of compound (69). In studies with TMCM, there was no significant difference in immune response *in vitro* of this compound compared to its diester counterpart (47), while studies with TDMs and TMMs revealed that the TDMs lead to greater cytokine production *in vitro*, yet the *in vivo* responses to both classes of glycolipids were similar (27).

It is difficult to explain why the Mincle agonist may give different immune profiles when tested *in vitro* or *in vivo*, and indeed, why differences might be observed upon subtle changes to *in vitro* models (e.g., plate-coated glycolipids compared to glycolipids in solution). To further illustrate this point, a C18 lipidated brartemicin derivate was found to give a similar immunomodulatory profile to TDB in several types of *in vitro* assay, yet the brartemicin derivative exhibited enhanced *in vivo* adjuvanticity in vaccination studies using ovalbumin as a model antigen (62). Conversely, a representative GMM was observed to lead to similar levels of cytokine production by BMMs when compared to cytokine production elicited by TDB, yet *in vivo*, GMM exhibited lower immunostimulatory potential than TDB (27). Moreover, computational docking and binding studies can assist in the rational design of Mincle agonists (77), although studies with brartemicin derivatives suggest that the strength of Mincle-binding may not always correlate to a functional immune response (62). Taken together, these findings suggest that *in silico* and *in vitro* profiling are useful tools to screen libraries of potential Mincle agonists but point to a need for several *in vitro* assays, with ultimately an *in vivo* model being performed to better determine the potential of lead Mincle agonists.

In addition to carbohydrate-derived Mincle agonists, there are also a number of cholesterol derivatives that activate Mincle, with plate-coated and crystalline cholesterol (and some derivatives thereof) being found to activate hMincle (but not mMincle or rMincle) (20), while cholesterol sulfate was shown to be an agonist for mMincle (95). In the former studies, cholesterol was determined to bind to the CRAC motif in Mincle, while the binding site of cholesterol sulfate was not determined. These studies also highlight potential species-specific activity across different classes of Mincle ligand. While Mincle has high sequence homology across a variety of species (1), with many classes of ligands (e.g., trehalose glycolipids) activating different Mincle homologs, species-specific activity has been observed in studies with GroMM (84) and with CMA esters of glycerol (47), which are ligands for hMincle but not mMincle, in addition to the aforementioned studies with cholesterol (20).

In summary, much has been discovered in the less than 10 years since TDM was identified as the first non-proteinaceous Mincle ligand, with many classes of glycolipids subsequently being determined to be Mincle agonists. Several lipids and cholesterol derivatives have also been found to be Mincle agonists. Thus, the potential scope for Mincle agonists appears vast, with some ligands already exhibiting promise as vaccine adjuvants or immunotherapeutics. Nevertheless, much remains unanswered in terms of better understanding the key structural features required for Mincle binding and activation. This, in turn, will require the continued collaboration between synthetic and computational chemists, immunologists, and microbiologists, so that the potential of Mincle ligands for the prevention or treatment of disease can best be realized.

## Author Contributions

All authors contributed to the writing and editing of this manuscript.

## Conflict of Interest Statement

The authors declare that the research was conducted in the absence of any commercial or financial relationships that could be construed as a potential conflict of interest.

## Abbreviations

APCs, antigen-presenting cells; AraMM, arabinosyl monomycolate; BMDCs, bone marrow-derived dendritic cells; bMincle, bovine Mincle; BMMs, bone marrow-derived macrophages; CLRs, C-typecalcium-dependent, lectin receptors; CMA, corynomycolic acid; CRAC, cholesterol recognition/interaction amino acid consensus; CRD, carbohydrate recognition domain; DAMPs, damage-associated molecular patterns; DDA, dimethyldioctadecylammonium; ELISA, enzyme-linked immunosorbent assay; GBA1, β-glucosylceramidase; β-GlcCer, β-glucosylceramide; Glc-DAG, glucosyl diacylglycerol; GMB, glucose monobehenate; GMCMs, glucose monocorynomycolates; GMMs, glucose monomycolates; GroMB, glycerol monobehenate; GroMCM, glycerol monocorynomycolate; GroMM, glycerol monomycolate; hMincle, human Mincle; IFA, incomplete Freund’s adjuvant; Ig, immunoglobulin; IL, interleukin; MCL, macrophage C-type lectin; Mincle, macrophage inducible C-type lectin; MIP-2, macrophage inflammatory protein 2; mMincle, mouse Mincle; moMφ, monocyte-derived macrophage; NF, nuclear factor; NFAT-GFP, nuclear factor of activated T cells-green fluorescent protein; PAMPs, pathogen-associated molecular patterns; rMincle, rat Mincle; SPR, surface plasmon resonance; TDB, trehalose dibehenate; TDCMs, trehalose dicorynomycolates; TDEs, trehalose diesters; TDM, trehalose dimycolate; TLRs, toll like receptors; TMCMs, trehalose monocorynomycolates; TMEs, trehalose monoesters; TMMs, trehalose monomycolates; TNF, tumor necrosis factor; WT, wild type.
